# Guarantees on the structure of experimental quantum networks

**DOI:** 10.1038/s41534-024-00911-z

**Published:** 2024-11-14

**Authors:** Andrés Ulibarrena, Jonathan W. Webb, Alexander Pickston, Joseph Ho, Alessandro Fedrizzi, Alejandro Pozas-Kerstjens

**Affiliations:** 1 Institute of Photonics and Quantum Sciences, School of Engineering and Physical Sciences, Heriot-Watt University, Edinburgh, UK; 2 Group of Applied Physics, University of Geneva, Geneva, Switzerland; 3 Instituto de Ciencias Matemáticas (CSIC-UAM-UC3M-UCM), Madrid, Spain

**Keywords:** Quantum information, Optics and photonics

## Abstract

Quantum networks connect and supply a large number of nodes with multi-party quantum resources for secure communication, networked quantum computing and distributed sensing. As these networks grow in size, certification tools will be required to answer questions regarding their properties. In this work we demonstrate a general method to guarantee that certain correlations cannot be generated in a given quantum network. We apply quantum inflation methods to data obtained in quantum group encryption experiments, guaranteeing the impossibility of producing the observed results in networks with fewer optical elements. Our results pave the way for scalable methods of obtaining device-independent guarantees on the network structure underlying multipartite quantum protocols.

## Introduction

As quantum information processing matures, there is an increased demand for certifying hardware devices or the underlying quantum resource with minimal assumptions. It is now possible to guarantee quantum phenomena from just statistics corresponding to few, uncharacterized measurements, using the device-independent framework^1,2^. Despite the amount of information from the system being minimal, the device-independent formalism allows, in certain situations, not just to guarantee that the device under scrutiny is quantum but to certify the quantum state and measurements that are being performed on it^3^, and properties such as nonlocality^2^, entanglement^4,5^, randomness^6^, the dimension of the underlying quantum state^7^, quantum measurements^8,9^, or superpositions of causal orders^10^.

The focus of device-independent quantum certification has been moving towards complex networks that feature several independent quantum systems being distributed to multiple parties^11^. In analogy with the device-independent certification methods in bipartite scenarios—which follow the spirit of Bell’s theorem^12^—many Bell-like inequalities have been developed that are satisfied by all correlations that can be generated in specific networks when the sources distribute classical systems^13–22^, and some of them have been found not to be satisfied in experiments^23–27^. Moreover, the device-independent analysis of the limits of quantum mechanics in networks has led to the demonstration of the necessity of complex numbers in order to account for all quantum correlations observed in nature^28,29^.

In this work, we take an orthogonal approach to certification in quantum networks. Typically, one assumes that the network structure underlying the setup under study is fixed, and non-compliance with the corresponding constraints signal the use of supra-classical^13,30,31^ or supraquantum^32^ resources. In contrast, we focus on providing guarantees on the network structure underlying some observations. As quantum networks become commonplace and more complex, it is vital to develop efficient and scalable tools that allow users to guarantee their integrity, including the network structure itself (which could be modified by colluding parties or by eavesdroppers), in order to correctly perform quantum protocols on them, safe from eavesdrops. In fact, it is already known that being able to guarantee a particular network structure allows certain, otherwise impossible^33^, cryptographic protocols^34^.

In order to provide such certifications on the structure of quantum networks, we assume that quantum mechanics accurately describes natural phenomena and thus there are no supraquantum resources in nature. In this case, observing a behavior beyond the limits of what is allowed by quantum mechanics in a given network is a demonstration that the actual network that is implemented is a different one. In this sense, the goal of our work aligns with that of refs. ^35–38^, having the additional hypothesis that quantum mechanics accurately describes nature. The recent work^39^ addresses this problem under the additional assumptions that the sources distribute states (close to) Greenberger-Horne-Zeilinger states and each party receives just one qubit. In contrast, the approach we describe in this work only assumes the validity of quantum mechanics and the independence of the sources of quantum systems in the network, thus taking a full device-independent perspective.

**Fig. 1:**
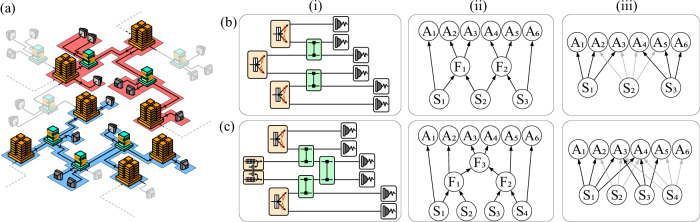
Testing the network structure in uncharacterized realizations of quantum networks. **a** A prototype quantum network: the golden, tall buildings represent factories of quantum states, which are distributed either to end-users (gray, small detectors) or “data centers” (green, medium buildings) that are allowed to perform arbitrary joint transformations on all the systems received. Our goal is to test the network structure just from observational data in the gray nodes. We illustrate the method in two networks: The process for the red shaded network is elaborated in row (**b**) and detailed in the main text, while the network in the blue region is discussed in row (**c**) and in the Supplementary Materials. Column (i) depicts specific realizations implemented in the corresponding networks, using circuit notation (i.e., with all the sources, gates and measurements). Column (ii) depicts the causal diagrams corresponding to the networks, where the sources *S*_*i*_ send systems to interact with each other in the nodes *F*_*i*_, and subsequently are distributed to the parties *A*_*i*_ which measure them. Column (iii) depicts network structures (with states and measurements, but no operations) that will be used to attempt to reproduce the correlations generated in the structures in column (ii). Our procedure consists in finding the most general network (column (iii)) with as many sources and parties as the realization (columns (i)-(ii)), characterizing the observations that can be produced in it, and demonstrating that the measurement statistics produced in the experiment do not belong to this set.

Our approach consists of, given some observations coming from an uncharacterized realization (e.g., columns (i-ii) in Fig. 1) proposing candidate network structures for their realization (e.g., column (iii) in Fig. 1), and test whether the candidate structure can generate the observations. Our goal will be to prove that the observations are incompatible with the candidate network, this is, that there exist no quantum states (of any dimension) and measurements that, once distributed according to the network structure, reproduce the observations.

We illustrate this approach by analyzing data from the realization of six-photon experiments^40,41^ used in quantum conference key agreement (QCKA) protocols^42–47^. We do so for two reasons: First, quantum cryptography is an important application where, if security proofs assume a specific network structure, guaranteeing this structure experimentally becomes crucial to discard potential eavesdrops^48^. Second, device-independent certification in QCKA protocols is achieved by contrasting the observed statistics against multipartite global local hidden variable models or by certifying the presence of genuine multipartite entanglement^49,50^. However, if the protocol is implemented in a given network, contrasting against the corresponding network models has advantages in terms of the requirements for certification (see, e.g.,^19,51^ for advantages in terms of the detection efficiency) in addition to those discussed above. Using readily available tools^52,53^, we will produce quantum Bell-like inequalities for particular hypotheses on the network structure underlying the experimental implementations and observe their violation by the empirical statistics.

## Results

### Necessary constraints on quantum network correlations

Characterizing the correlations that are generated in network scenarios is a notably hard problem. For some networks, there exist simple necessary conditions for correlations to be compatible: when two parties share no causal history, their joint distribution factorizes. This is the case, for instance, of parties *A*_1_ and *A*_6_ in Fig. 1(b–ii) and (b-iii), or between the extremal parties in the entanglement-swapping network^13^. However, there exist networks where no such factorizations appear. The simplest example is the triangle network, obtained from the entanglement-swapping network by adding a new source connecting the extremal parties. This network, being the simplest one where explicit factorizations fail to characterize it, has been subject to intense study^15,32,54–57^.

In these cases without explicit factorizations, one can derive necessary conditions for correlations compatible with a network by means of inflation^52,58^. Briefly, inflation allows to derive compatibility constraints by imagining that multiple copies of the sources distributing physical systems and of the measurement devices held by the parties are available, and analyzing the correlations that are obtained when connecting these copies. The network structure is reflected in symmetries in the correlations on the new, inflated networks, which are much simpler to enforce and analyze (see the Methods). Indeed, many of such constraints are now present in the literature, mostly in the form of Bell-like inequalities^15,54,57^. The inequalities provided by inflation methods are polynomial, i.e. of the form

1 $$\sum _{n}\sum _{\begin{array}{c}{\vec{a}}_{1}\ldots {\vec{a}}_{n}\\ {\vec{x}}_{1}\ldots {\vec{x}}_{n}\end{array}}{c}_{{\vec{a}}_{1},\ldots ,{\vec{a}}_{n},{\vec{x}}_{1},\ldots ,{\vec{x}}_{n}}p({\vec{a}}_{1}| {\vec{x}}_{1})\cdots p({\vec{a}}_{n}| {\vec{x}}_{n})\ge 0,$$

where ${c}_{{\vec{a}}_{1},\ldots ,{\vec{a}}_{n},{\vec{x}}_{1},\ldots ,{\vec{x}}_{n}}$ are real coefficients, each ${\vec{a}}_{i}$ is a vector of outputs obtained by all parties, and ${\vec{x}}_{i}$ is the vector of corresponding inputs. These inequalities are derived naturally from the separating hyperplanes that appear when solving the linear or semidefinite programs associated to, respectively, classical^58^ and quantum^52^ inflation problems in any network. Finding for some $p(\vec{a}| \vec{x})$ that the left-hand side evaluates to a negative quantity is a guarantee that $p(\vec{a}| \vec{x})$ does not admit the quantum inflation used to produce the inequality. Importantly, admitting an inflation of a network is a relaxation of admitting a realization in said network. Therefore, detecting that some correlations do not admit an inflation of a network is a proof that they cannot be produced in the original network.

In order to illustrate the full method that we propose, let us consider the experimental realization that is used to produce six-partite Greenberger-Horne-Zeilinger (GHZ) states in ref. ^41^, illustrated in Fig. 1b. There, six-photon multipartite entangled states are created and distributed to six parties, *A*_1_, …, *A*_6_. Three photon-pair sources generate Bell pairs, and subsequently two fusion gates are used to obtain the final six-photon GHZ state. After the measurements, the outcome statistics follow the corresponding Born’s rule, namely

2 $$\begin{array}{rcl}p({a}_{1},\ldots ,{a}_{6})&=&\,\text{Tr}\,\left[{U}_{23}\otimes {U}_{45}\left({\phi }_{12}^{+}\otimes {\phi }_{34}^{+}\otimes {\phi }_{56}^{+}\right){U}_{23}^{\dagger }\otimes {U}_{45}^{\dagger }\right.\\ &&\left.\qquad \left({\Pi }_{{a}_{1}}\otimes \cdots \otimes {\Pi }_{{a}_{6}}\right)\right],\end{array}$$

where ${\phi }^{+}=\frac{1}{2}(\left\vert 00\right\rangle +\left\vert 11\right\rangle )(\left\langle 00\right\vert +\left\langle 11\right\vert )$ is the maximally entangled state, *U*_*ij*_ is the unitary implementing the fusion gate between photons *i* and *j*, and ${\Pi }_{{a}_{i}}$ are the projectors describing the measurement operator of party *A*_*i*_. One can consider distributions with inputs, *p*(*a*_1_, …, *a*_6_∣*x*_1_, …, *x*_6_) by using different projectors ${\Pi }_{{a}_{i}}^{{x}_{i}}$ for each measurement. For more details on the six-photon state generation, we refer to the Supplementary Materials and the original reference^41^.

The first step in the procedure is finding a network (i.e., a bipartite graph representation that only contains sources and parties^11^) that closely resembles the structure of the experiment. This network will have as many sources and outcomes as the experimental realization. Each of the sources will distribute systems to all the parties which, in the experiment, have a causal connection to it. For the setup of Fig. 1b this means that the leftmost (respectively rightmost) source will distribute systems to the three leftmost (respectively rightmost) parties, and the central source will distribute systems to the four central parties, leading to the network in Fig. 1(b–iii). Distributions that are generated in this network take the form given by the corresponding Born’s rule, i.e.,

3 $$p({a}_{1},\ldots ,{a}_{6})=\,\text{Tr}\,\left[\left({\rho }_{{S}_{1}}\otimes {\rho }_{{S}_{2}}\otimes {\rho }_{{S}_{3}}\right)\cdot \left({\Pi }_{{a}_{1}}\otimes \cdots \otimes {\Pi }_{{a}_{6}}\right)\right],$$

where each ${\rho }_{{S}_{i}}$ represents an arbitrary state distributed by source *S*_*i*_.

In order to discern whether the quantum correlations generated in the structure in Fig. 1(b–i), (b–ii) via Eq. (2) can be reproduced in the network of Fig. 1(b–iii) via Eq. (3) we will use quantum inflation^52^ as described in the Methods (namely, by relaxing the problem to a hierarchy of semidefinite programs that test the existence of distributions on extended scenarios with appropriate symmetries and constraints over their marginals). This implies, in particular, that we impose no restriction on the dimension of the systems distributed by the sources nor in the measurements that the parties perform on all the shares of their respective systems. We thus allow to create strong correlations between the systems in the network in Fig. 1(b–iii). Yet, we will show that these are not strong enough to reproduce the multi-photon correlations observed in Fig. 1(b–i).

In the remainder of the manuscript we focus on analyzing conditions that correlations that can be generated in the network of Fig. 1(b–iii) and how the experimental data produced in Fig. 1(b–i) (found in Ref. ^41^) does not meet them, showcasing the importance of the fusion gates in the realization. We must stress that our approach is fully general, not restricted to the setup in Fig. 1(b–i). In order to illustrate the generality of the approach, in the Supplementary Materials we perform an analogous analysis for the experiment carried out in ref. ^40^, depicted in Fig. 1c.

### Witnesses of network incompatibility

When using quantum inflation, witnesses of incompatibility can be obtained in a direct manner, exploiting the fact that the compatibility of a distribution with a quantum inflation can be formulated as a semidefinite program^52,59^. These are optimization problems that, upon finding an incompatible distribution (recall, one for which no quantum states and measurement operators exist that reproduce it in the candidate network), provide a witness in the form of Eq. (1) that is positive for all compatible distributions and evaluates negatively at least for the incompatible one. Importantly, its evaluation to a negative quantity by any distribution is a guarantee that such distribution does not admit a realization in the candidate network.

We will obtain these witnesses for several distributions of the form of Eq. (2). Then, as a second step, we will evaluate the witnesses on the empirical data obtained in ref. ^41^. In order to do so, we convert the raw counts from the detectors into a probability distribution of six-photon events. The experimental setup employs measurement stages with two outputs, denoted by the transmission of a horizontally polarised photon through the polarising beam splitter or the reflection of a vertically polarised one. By normalising the number of six-photon counts (one per party) obtained in each of the possible events by the total number of six-photon counts, we obtain the empirical distributions that we will test.

#### Binary-input distribution

The data in ref. ^41^ contains counts for all the parties measuring in the *X* and *Z* bases. Therefore, it is possible to consider the two-input distribution *p*(*a*_1_, …, *a*_6_∣*x*_1_, …, *x*_6_), where *x*_*i*_ = 0 corresponds to the measurement on the *X* basis and *x*_*i*_ = 1 corresponds to the measurement on the *Z* basis. We find the resulting theoretical distribution (2), using ideal states and measurements, not to admit a realization in the network of Fig. 1(b–iii) (i.e., an expression of the form of Eq. (3)) by using its corresponding second-order inflation (depicted in Fig. 4) and, already, at the first level of the associated Navascués-Pironio-Acín (NPA) hierarchy^60,61^. The hierarchy is defined via sets of operators ${{\mathcal{O}}}_{n}$ that index the rows and columns of the matrix ${\Gamma }_{i,j}^{n}=\,\text{Tr}\,[\rho \cdot {O}_{i}^{\dagger }{O}_{j}]$. If a distribution admits a quantum realization, *Γ*^*n*^ is positive semidefinite for any generating set ${{\mathcal{O}}}_{n}$, and if it does not there exists at least one ${{\mathcal{O}}}_{n}$ for which *Γ*^*n*^ is negative definite. The first level of the hierarchy, which we use for analyzing the two-input distribution, is defined by the set of operators ${{\mathcal{O}}}_{1}:=\{{\mathbb{1}}\}\cup \{{A}_{p}^{i,j}\}$, leading to a matrix of size 41 × 41 in our case of interest, whose positivity can be determined in <1 s. In the Supplementary Materials we elaborate further on the details of the implementation.

The guarantee of incompatibility is given by the witness in Eq. S1 in the Supplementary Materials (see also the computational appendix^62^ for the code executed to obtain it), extracted from the problem. This witness does not only identify Eq. (2) as incompatible, but when adding white noise to the maximally entangled states, ${\phi }_{ij}^{+}\mapsto v{\phi }_{ij}^{+}+(1-v){\mathbb{1}}/4$, it identifies that the distribution is incompatible at least for *v* ≳ 0.6180. By increasing the accuracy of the approximations of Eq. (3) provided by inflation (see the Supplementary Materials) it is possible to guarantee incompatibility for, at least, all *v* ≳ 0.3887.

**Fig. 2:**
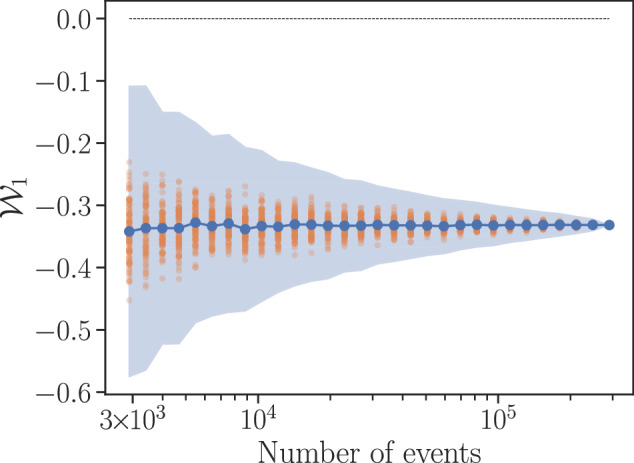
Evaluation of the structure witness in Eq. S1 in the experimental data of ref. ^41^. The maximum value achievable by quantum distributions generated in the network of Fig. 1(b–ii) is upper bounded by 0, so evaluating to a negative number by a given distribution is a witness that such distribution cannot be generated in Fig. 1(b–iii). In the horizontal axis we denote the amount of all datapoints, chosen at random, used for computing the witness. Error bars correspond to five standard deviations over 100 repetitions. The individual results are depicted by the orange points. The magnitude of the witness gives a notion of the distance to the set of compatible distributions, but in general it lacks of a concrete physical meaning.

The evaluation on the experimental data is shown in Fig. 2. Notably, even the smallest amount of data considered, namely ~ 2900 six-photon events, representing 1% of the total amount, allows for a robust violation (namely ${{\mathcal{W}}}_{1}=-0.3420\pm 0.0466$) well beyond the five-deviation limit. This represents an acquisition time of only 60 s per measurement basis, or equivalently, a total use of the experiment of ~1 h. In combination with the key generation results of ref. ^41^, this result demonstrates that it is feasible to dedicate a small amount of the data for certifying the network structure and use the rest to establish key at significantly higher rates than via concatenations of bipartite protocols.

We also analyze the data of ref. ^40^, corresponding to the network in Fig. 1c, in the Supplementary Materials. In this case, the available data allows for obtaining three binary-input distributions, corresponding to the parties performing their measurement along the bases {*X*−*Y*, *X*−*Z*, *Z*− *Y*}. We are able to obtain inequalities that witness incompatibility for all distributions for *v* ≳ 2^−1/4^. However, the differences in time spent accumulating six-photon coincidences per measurement basis (~5 min for ref. ^40^, totalling ~1000 six-photon events per basis, versus ~3.5 h for Fig. 1(b–i), totalling ~ 4500 six-photon events per basis), reflect themselves in the fact that the empirical distributions are not witnessed incompatible in the former case.

#### Tests for no-input distributions

**Fig. 3:**
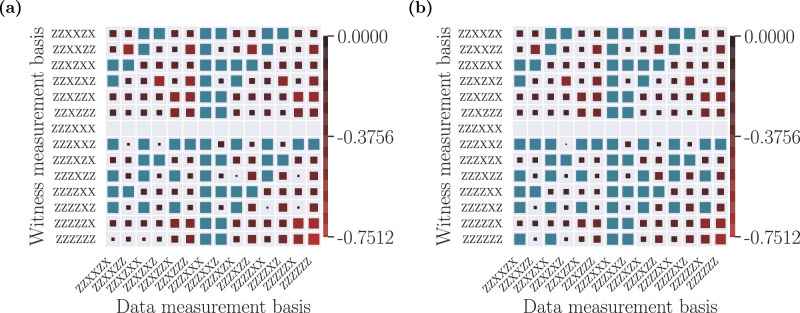
Witnesses of incompatibility for no-input distributions. **a** Theoretical predictions and (**b**) experimental results for no-input witnesses of incompatibility with the network of Fig. 1(b–iii). The indexing of the rows and columns denotes the measurement operators that are used to generate a no-input probability distribution according to Eq. (2). Using the procedure described in the text, the distributions denoted by the rows are found to be incompatible with realizations in the network of Fig. 1(b–iii), each one producing a witness of incompatibility. Then, each witness is evaluated on all distributions denoted by the columns, producing the figures where each cell represents the evaluation of the witness obtained from the distribution in the row in the distribution in the column. The blue cells denote distributions that are not detected by a particular witness, i.e. those that evaluate to a positive value. The empty rows denote ideal distributions that are not detected to be incompatible with the inflation used. The size and the color of the red squares denote the strength of the detection for distributions witnessed to be incompatible. The complete figures with the 64 possible input combinations can be found in Figs. S1, S2 on the Supplementary Materials, and a selection of the value of the inequalities as a function of the amount of data used can be found in Fig. S3.

Certifying quantum properties, such as non-locality or entanglement, in a device-independent manner necessitates of the parties performing different measurements on the shares of the states received. In contrast, it is known that constraints on the network structure are encoded even on distributions without inputs^54^, i.e., when the parties do not have a choice of measurement but they always measure the same operator in the received shares. One can therefore use no-input distributions to attempt at extracting guarantees of the network structure. This reduces the amount of data needed for the certification: while in the binary-input case one needs all the 2^12^ probabilities *p*(*a*_1_, …, *a*_6_∣*x*_1_, …*x*_6_) for *a*_1_, …, *a*_6_, *x*_1_, …*x*_6_ ∈ {0, 1}, using no-input distributions needs only of *n* ⋅ 2^6^ probabilities ${\{{p}_{k}({a}_{1},\ldots ,{a}_{6})\}}_{k = 1}^{n}$, where *n* is the total number of distributions tested. Therefore, in principle, one could use the techniques described earlier to give guarantees in the network structure even with fewer data. Unfortunately, this gain does not come for free. Any no-input distribution can always be simulated by a single source of shared randomness, and therefore any violation of a single inequality can always be attributed to an adversary classically correlating the parties’ outcomes. However, in the same way that the classical distribution $p(a,b,c)=\frac{1}{2}\,\,\text{if}\,\,a=b=c$ can simulate the correlations of measurements on the *Z* basis performed on the state $(\left\vert 000\right\rangle +\left\vert 111\right\rangle )/\sqrt{2}$ but not those of *X* measurements, having a distribution being detected by several witnesses tailored for different bases gives mounting evidence of its incompatibility with a quantum network. Therefore, in the following we will extract witnesses for multiple no-input distributions, and we will evaluate each distribution in all of them to understand which distributions are easier to detect as incompatible, in the sense that they violate the largest amount of witnesses. The main results for the state created in the network in Fig. 1(b–ii) are shown in Fig. 3. There, the color code denotes the value of the witness of incompatibility of the distribution obtained by measuring the state with the operators indicating the row with the network of Fig. 1(b–iii), when evaluated in the distribution obtained by measuring the state with the operators indicating the column. More complete figures, for all possible measurement bases, can be found in the Supplementary Materials, and all the witnesses found are stored in the computational appendix^62^.

**Fig. 4:**
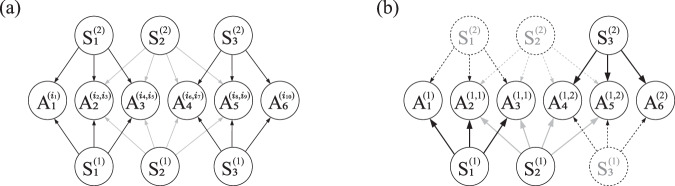
Illustration of inflation. **a** Second-order quantum inflation of the network in Fig. 1(b–iii). There are two copies of each of the sources, and each party now has access to a different copy of the original measurement operators for each combination of states they receive. The distributions ${p}_{\inf }({\{{a}_{1}^{{i}_{1}},{a}_{2}^{{i}_{2},{i}_{3}},\ldots ,{a}_{6}^{{i}_{10}}\}}_{{i}_{1},\ldots ,{i}_{10}})$ produced in this scenario have a number of symmetries and marginals fixed by the original distribution *p*(*a*_1_, …, *a*_6_). The sequence of operators in (**b**) illustrate an assignment of indices (*i*_1_ = *i*_2_ = *i*_4_ = 1, *i*_3_ = *i*_5_ = *i*_6_ = *i*_8_ = 1, *i*_7_ = *i*_9_ = *i*_10_ = 2) that reproduces the original network, and thus the corresponding marginals must reproduce *p*(*a*_1_, …, *a*_6_). The fact that a ${p}_{\inf }({\{{a}_{1}^{{i}_{1}},{a}_{2}^{{i}_{2},{i}_{3}},\ldots ,{a}_{6}^{{i}_{10}}\}}_{{i}_{1},\ldots ,{i}_{10}})$ that satisfies all the necessary symmetries and marginal constraints does not exist is a proof that the premise (recall, that *p*(*a*_1_, …, *a*_6_) can be generated in the network of Fig. 1(b–iii)) is not true. The existence of a suitable ${p}_{\inf }({\{{a}_{1}^{{i}_{1}},{a}_{2}^{{i}_{2},{i}_{3}},\ldots ,{a}_{6}^{{i}_{10}}\}}_{{i}_{1},\ldots ,{i}_{10}})$ is a problem that can be formulated in terms of semidefinite programming^59–61^.

Since the data in ref. ^41^ contains statistics for all measurement choices in {*X*, *Z*}^×6^, we analyze all such distributions, assessing again their compatibility with the second-order quantum inflation of the network in Fig. 1(b–iii), depicted in Fig. 4. We obtain witnesses of incompatibility for a total of 40 distributions. When evaluating them on the distributions resulting from considering that the sources distribute Werner states of visibility *v*, these witnesses allow to detect incompatibility for visibilities ranging between 0.7808 (for the distribution corresponding to measurements *XZZZZX*, that establishes key between the four central parties) to 0.4094 (for the distributions corresponding to measurements *ZXZZZZ*, *ZZXZZZ*, *ZZZXZZ* and *ZZZZXZ*, that establish key between five of the six parties). Then, we evaluate the witnesses on the distributions corresponding to all measurement bases in {*X*, *Z*}^×6^. In Fig. 3a, b we show, respectively, the theoretical predictions for the noiseless distributions of the form of Eq. (2) and the evaluations on the empirical data, for a subset of witnesses and distributions. Analogous plots for all the witnesses and distributions can be found in Figs. S1 and S2 in the Supplementary Materials. These figures show that many distributions are witnessed as incompatible by a significant amount of inequalities, giving mounting evidence to the impossibility of generating them in the network of Fig. 1(b–iii).

Remarkably, we observe a very good agreement between the theory and the experimental data. This is important given that the experimental data acquisition time per basis on a single experimental run was only 60 s and, over the course of several iterations, the data acquisition time totals ~ 3.5 hours per basis. On average, on a single run there are on the order of ~20 six-photon events. Moreover, as in the case of Fig. 2, the results are stable with regards to the amount of data used for computing the statistics. In Fig. S3 in the Supplementary Materials we illustrate this stability by plotting how a selected number of inequalities detect the incompatibility of several distributions. As we can see, even using only a fraction of the data we consistently obtain conclusive results and, when using ~2000 six-photon events, the uncertainty of the violation is below the five-sigma level. Again, we perform the equivalent analysis for the data of ref. ^40^, corresponding to the network in Fig. 1c, in the Supplementary Materials.

## Discussion

In this work we report on the first device-independent analysis on quantum network structure of experimental implementations. We generated Bell-like inequalities with quantum inflation^52,53^, a general method that can be used with any network configuration. Using the statistics from real experiments, we ruled out the possibility that these are generated in alternative networks that contain fewer interactions despite allowing for larger physical systems. The fact that the procedure generates Bell-like inequalities implies that the witnesses obtained can be applied to arbitrary distributions, thus not being restricted to the experiments under scrutiny in this work.

A distribution being detected by a witness is a guarantee that such distribution cannot be generated in the corresponding network, but not being detected does not imply that the distribution can be generated in the network. Indeed, it is possible that using more constraining inflations leads to stronger witnesses. However, this incurs in additional computational load, which quickly goes beyond standard available resources. In the six-photon realizations that we have tested, we were able to solve each of the necessary optimization problems in less than 2.5 minutes in a laptop. However, going beyond an inflation level of 2 (i.e., considering two copies of each of the sources in the realization) is beyond standard computational capabilities, both memory- and time-wise.

It is possible to find violations of the inequalities using classical resources in more general networks. For instance, many of the no-input inequalities are violated by a uniformly random bit shared among all the parties, which necessitates of a six-partite source. Adding the ability of the parties to perform different measurements to the systems they receive may alleviate the issue. Obtaining witnesses that do not detect classical realizations in more general networks is an important topic that is left to future work.

Other techniques for analyzing network structure currently exist in the literature, that are based on the analysis of entropies^63^ or covariances^64^ of the observations. However, these typically focus exclusively in the constraints implied by the network structure, and thus produce criteria that are satisfied even by non-quantum distributions, if they are generated according to the structure dictated by the network. In this sense, quantum inflation becomes the most suitable tool, since it allows to take into account both the network structure and the fact that the systems distributed are quantum, and the strength of both types of constraints can be tuned independently [ref.^65^, Ch. 5].

The process described is completely general, applicable to any experimental scenario and protocol. On a more practical side, the procedure developed in this work can be used for estimating critical values for the experimental requirements of protocols, in a spirit similar to that of ref. ^55^. This is especially important as the traditional loopholes associated to device-independent protocols are closed, which comes at the cost of more demanding experimental requirements^66^. Finally, it is possible to define, with the same computational requirements, stronger characterizations with inflation that the ones we have used here by considering the so-called *linearized polynomial identification* relations^57^, at the expense of not being able to obtain inequalities that can detect the infeasibility of other distributions.

We applied the procedure to the analysis of concrete realizations relevant in quantum conference key agreement, showing that the data used for generating key can be recycled to also provide guarantees on the network structure. This provides a strong motivation for developing of novel quantum information protocols tailored to networks, which is currently a largely unexplored field^48^. Other applications of the methods outlined in this paper could involve the verification of networks generated in programmable photonic chips, such as the ones in ref. ^67^. However, in this case one must bear in mind the difficulty of closing the locality loophole.

## Methods

### Inflation methods for obtaining witnesses of incompatibility

The witnesses of network structure that are obtained in this work are calculated using the inflation technique. For simplicity in the notation, we present here the main ideas behind inflation methods for the case of distributions without inputs. Adding inputs to the construction can be done in the trivial way. For more in-depth discussions, we refer the reader to the original works^52,58^, the corresponding sections in the reviews^11,59^, or the appendices of refs. ^21,57^.

In order to demonstrate that a particular distribution cannot be generated in a given (in our case of study, quantum) network, inflation uses a strategy of reduction to the absurd: the fact that a distribution is compatible with a quantum network implies that there exist quantum states and measurement operators that reproduce it. If such is the case, one can consider the (hypothetical) situation where access is provided to multiple copies of said states and operators, and analyze the distributions of outcomes that are produced when these are arranged in more complicated networks.

As an illustration of how the inflation reasoning works, Fig. 4 depicts the inflation of the network in Fig. 1(b–iii) that we use throughout the work, where we consider two copies of each of the sources of quantum systems in the original network. The sources ${S}_{i}^{(k)}$ are all copies of the original source *S*_*i*_, and the operators ${A}_{p}^{i,j}$ denote copies of the original measurement operators, *A*_*p*_, that are applied on copies *i* and *j* of the corresponding sources. For simplicity, let us consider the case where all parties do not have a choice of measurement to perform, and that the measurements have binary outcomes. Note that the arguments below easily carry over to the cases of more measurements and outcomes. If a probability distribution *p*(*a*_1_, …, *a*_6_), *a*_1_, …, *a*_6_ ∈ {0, 1} admits a realization in the network of Fig. 1(b–iii), i.e., if there exist states delivered by the sources *S*_1_, *S*_2_, and *S*_3_, and measurement operators *A*_1_, …, *A*_6_ that reproduce said distribution via Eq. (3) (with the projectors defined as ${\Pi }_{{a}_{i}}=\frac{1}{2}\left({\mathbb{1}}+{(-1)}^{{a}_{i}}{A}_{i}\right)$), then one can easily consider copies of those states and measurements to produce a distribution, ${p}_{\inf }({\{{a}_{1}^{{i}_{1}},{a}_{2}^{{i}_{2},{i}_{3}},\ldots ,{a}_{6}^{{i}_{10}}\}}_{{i}_{1},\ldots ,{i}_{10}})$, in the arrangement depicted in Fig. 4a.

The structure of Fig. 4a imposes a number of properties for the distributions ${p}_{\inf }({\{{a}_{1}^{{i}_{1}},{a}_{2}^{{i}_{2},{i}_{3}},\ldots ,{a}_{6}^{{i}_{10}}\}}_{{i}_{1},\ldots ,{i}_{10}})$ that can be generated in it. First, since all ${S}_{i}^{(k)}$ represent exact copies of the source *S*_*i*_, expectation values of arbitrary polynomials of the measurement operators are invariant under permutation of the sources. As an illustration (but not limited to it), one of such properties is that ${p}_{\inf }({\{{a}_{1}^{{i}_{1}},{a}_{2}^{{i}_{2},{i}_{3}},\ldots ,{a}_{6}^{{i}_{10}}\}}_{{i}_{1},\ldots ,{i}_{10}})={p}_{\inf }({\{{a}_{1}^{\pi ({i}_{1})},{a}_{2}^{\pi ({i}_{2}),{\pi }^{{\prime} }({i}_{3})},\ldots ,{a}_{6}^{{\pi }^{{\prime\prime} }({i}_{10})}\}}_{{i}_{1},\ldots ,{i}_{10}})$, where $\pi ,{\pi }^{{\prime} }$ and *π″* are independent permutations of the sources *S*_1_, *S*_2_ and *S*_3_, respectively. Second, when one restricts to marginals that reproduce the original network (or parts of it), these coincide with the original distribution under scrutiny. This is, ${p}_{\inf }({a}_{1}^{i},{a}_{2}^{i,j},{a}_{3}^{i,j},{a}_{4}^{j,k},{a}_{5}^{j,k},{a}_{6}^{k})=p({a}_{1},\ldots ,{a}_{6})$ for any values of *i*, *j*, *k*. For instance, the case for *i* = 1, *j* = 1, *k* = 2 is depicted in Fig. 4b. Note that these are properties of any ${p}_{\inf }({\{{a}_{1}^{{i}_{1}},{a}_{2}^{{i}_{2},{i}_{3}},\ldots ,{a}_{6}^{{i}_{10}}\}}_{{i}_{1},\ldots ,{i}_{10}})$ that can be generated in the network in Fig. 4 if the premise that *p*(*a*_1_, …, *a*_6_) can be generated in the network in Fig. 1(b–iii) is true. Therefore, a demonstration that no such ${p}_{\inf }({\{{a}_{1}^{{i}_{1}},{a}_{2}^{{i}_{2},{i}_{3}},\ldots ,{a}_{6}^{{i}_{10}}\}}_{{i}_{1},\ldots ,{i}_{10}})$ exists is a proof that *p*(*a*_1_, …, *a*_6_) is incompatible, meaning that no quantum states and measurement operators exist that reproduce *p*(*a*_1_, …, *a*_6_) when used in the network of Fig. 1(b–iii).

In fact, one can relax the problem above to not consider distributions that can be generated in the inflated network of Fig. 4, but to consider distributions that just present the required symmetries and marginals. The set of such distributions is potentially more general, and in no case more restricted than that of the distributions that are achievable in Fig. 4. Moreover, in the case of quantum distributions, this relaxed set can be characterized by a hierarchy of semidefinite programming problems, each of which is more restrictive than the next one and all of which contain the original set^59–61^. This has two consequences that are particularly useful in our case of interest. First, it gives a collection of tests which can be efficiently performed, and that guarantee that *p*(*a*_1_, …, *a*_6_) cannot be generated in the network if any one of them fails. Second, if a distribution cannot be generated in a given quantum network, and this is detected by one of the semidefinite programs in the hierarchy, it is possible to derive from this a hyperplane that separates the distribution from the set of those that pass the test. This hyperplane is, thus, an incompatibility witness or a Bell-like inequality, that is satisfied by all compatible distributions and violated, at least, by the target distribution. Importantly, since it is the property that provides this technique of its power in terms of certification, the violation of the inequality by any distribution is a witness of incompatibility of such distribution with the quantum network. The opposite, namely that the inequality is satisfied, is not a guarantee of compatibility, since it could be possible that taking higher steps in the hierarchy leads to an inequality that is violated.

## Supplementary information

Supplemental Material

## Data Availability

No new data was generated in this work. The data used for generating Figs. 2 and 3b, and Figures S2, S3, S5b, and Table I in the Supplementary Materials is taken from refs. ^40,41^.

## Code availability

All the code used for generating the figures in this manuscript are available at ref. ^62^.
