# Supplementary material for: Guarantees on the structure of experimental quantum networks
Source: npj Quantum Inf. 2024 Nov 14;10(1):117. doi: 10.1038/s41534-024-00911-z (PMC11564111; doi:10.1038/s41534-024-00911-z)
Supplement: Supplementary file 1 — Supplemental Material [file 41534_2024_911_MOESM1_ESM.pdf]

Supplementary Material for "Guarantees on the structure of experimental quantum networks"

Andrés Ulibarrena,[1] Jonathan W. Webb,[1] Alexander Pickston,[1]
Joseph Ho,[1] Alessandro Fedrizzi,[1] and Alejandro Pozas-Kerstjens[2,3,4]

[1] *Institute of Photonics and Quantum Sciences, School of Engineering and Physical Sciences, Heriot-Watt University, Edinburgh EH14 4AS, United Kingdom*

[2] *Group of Applied Physics, University of Geneva, 1211 Geneva 4, Switzerland*

[3] *Constructor Institute of Technology, 8200 Schaffhausen, Switzerland*

[4] *Instituto de Ciencias Matemáticas (CSIC-UAM-UC3M-UCM), 28049 Madrid, Spain*

EXPERIMENTAL SETUP

The experimental realizations from which we use data are described in detail in the corresponding references, namely [41] for the setup in Fig. 1(b) and [40] for the setup in Fig. 1(c). In both, a Ti:Sapph pulsed laser, with a central frequency at 774.9 nm, feeds type-II parametric down-conversion (PDC) sources (which correspond to the leftmost boxes on Fig. 1(i) in the main text). The sources employ aperiodically poled Potassium Titanyl Phosphate (apKTP) crystals, which can produce two-photon interference visibilities up to $98.6 \pm 1.1\%$ [68], even without filtering the photons afterwards. Each source can be set to produce either maximally entangled photon pairs (these are denoted by the orange ∞ symbols), separable pairs (no symbol), or any intermediate configuration.

So as to create larger networks, individual photons from two different pairs are combined in type II fusion gates [69] (denoted by the boxes connecting two different photon sources on Fig. 1). After the required photons are fused, each photon is sent to a tomography stage (rightmost boxes in Fig. 1). Postselection on successful events, i.e. detecting a photon in one detector per tomography stage, is performed. Both the source configuration and the number and placement of the fusion gates reflects on the causal structure of the final state, as can be seen by comparing Figs. 1 (i), (ii) and (iii).

INFLATION METHODS FOR OBTAINING WITNESSES OF INCOMPATIBILITY

The witnesses of network structure that are obtained in this work are calculated using the inflation technique [52, 58]. In order to demonstrate that a particular distribution cannot be generated in a given (in our case of study, quantum) network, inflation uses a strategy of reduction to the absurd: the fact that a distribution is compatible with a quantum network implies that there exist quantum states and measurement operators that reproduce it. If such is the case, one can consider the (hypothetical) situation where access is provided to multiple copies of said states and operators, and analyze the distributions of outcomes that are produced when these are arranged in more complicated networks, as described in the main text or, for instance, in Refs. [11, 52, 57-59]. In the analysis of quantum distributions in networks, the study of these distributions in more complicated networks can be done by means of semidefinite programming. This means that, upon detecting a distribution that is incompatible with the constraints derived from the network, it is possible to extract a witness of such incompatibility. This witness can then be used to identify other distributions as incompatible, this is, it is a Bell-like inequality, satisfied by all compatible distributions and violated by at least some incompatible ones.

In order to obtain the Bell-like inequalities, we consider the distributions that are created when, in the implementation of Fig. 1(b-i), the sources distribute Werner states, $\rho_v = v\left|\phi^+\right\rangle\left\langle\phi^+\right| + (1-v)\mathbb{1}/4$, where $\left|\phi^+\right\rangle = (\left|00\right\rangle + \left|11\right\rangle)/\sqrt{2}$ is the maximally entangled state. For simplicity, we assume the same visibility, v, for all sources. We compute the smallest value of v such that the observed correlations do not admit an inflation, and for that distribution we extract the corresponding certificate of infeasibility. This takes the form of a polynomial Bell-like inequality, which we evaluate on the experimental data in order to detect that these, created in the setup in Fig. 1(b-i) in the main text, could not have been produced in the network of Fig. 1(b-iii). All this procedure is written using the `inflation` library [53] and the associated codes are available in the computational appendix [62].

Witnesses of network structure for binary-input distributions

The first case we deal with is the family of distributions $p(a_1,\ldots,a_6|x_1,\ldots,x_6)$ that is generated when the parties measure either σ_X or σ_Z on their respective photons in the setup of Fig. 1(b-i) in the main text. We will prove that such distribution cannot be reproduced in the quantum network of Fig. 1(b-iii), without constraining neither the dimension of the states distributed by the sources nor the measurements that the parties perform on the shares they receive. We do so by showing that $p(a_1,\ldots,a_6|x_1,\ldots,x_6)$ does not admit an inflation distribution in the inflation depicted in Fig. 2 in the main text. For this, we use the first level of the associated Navascués-Pironio-Acín hierarchy of semidefinite programs [60, 61], which is described in the main text. The hierarchy is defined via sets of operators $\mathcal{O}_n$ that index the rows and columns of the matrix $\Gamma^n_{i,j} = \mathrm{Tr}[\rho\cdot O_i^\dagger O_j]$. If a distribution admits a quantum realization, Γ^n is positive semidefinite for any generating set $\mathcal{O}_n$, and if it does not there exists at least one $\mathcal{O}_n$ for which Γ^n is negative definite. The first level of the hierarchy is defined by the set of operators $\mathcal{O}_1 := \{\mathbb{1}\}\cup\{A_p^{i,j}\}$, leading to a matrix of size 41×41 in our case of interest.

When we assume that all the sources in Fig. 1(b-i) distribute Werner states of some visibility v, $\rho_v = v\,|\phi^+\rangle\langle\phi^+| + (1-v)\frac{\mathbb{1}}{4}$ with $|\phi^+\rangle = (|00\rangle+|11\rangle)/\sqrt{2}$ being the maximally entangled state, the matrix Γ^1 cannot be made positive semidefinite for $v \gtrsim 0.6180$. This means that, at least for $v \gtrsim 0.6180$, it is not possible to reproduce the correlations generated in Fig. 1(b-i) (i.e., those of the form given by Eq. (3) in the main text) in the network of Fig. 1(b-iii) (i.e., in the form of Eq. (1) in the main text). This incompatibility is witnessed by the following inequality:

$$\begin{aligned}\mathcal{W}_1 :=\, & p_B(0)+p_C(0)+p_D(0)+p_E(0)+0.474\,[p_A(0)+p_F(0)]-0.886p_A(0)p_F(0)\\ & -0.768\,[p_{AB}(0,0)+p_{AC}(0,0)+p_{DF}(0,0)+p_{EF}(0,0)]+0.051\,[p_A(0)\,(p_B(0)+p_C(0))+(p_D(0)+p_E(0))\,p_F(0)]\\ & -0.758\,[p_{BC}(0,0)+p_{DE}(0,0)]-0.122\,[p_B(0)p_C(0)+p_D(0)p_E(0)]\\ & -0.621\,[p_{BD}(0,0)+p_{BE}(0,0)+p_{CD}(0,0)+p_{CE}(0,0)]+0.041\,[p_C(0)+p_B(0)]\,[p_D(0)+p_E(0)]\\ & +0.717\,[(p_B(0)+p_C(0))\,p_F(0)+p_A(0)\,(p_D(0)+p_E(0))]\\ & -0.031\left[p_A(0)^2+p_F(0)^2\right]+0.02\left[p_B(0)^2+p_C(0)^2+p_D(0)^2+p_E(0)^2\right],\end{aligned} \tag{S1}$$

where $p_{p_1p_2}(x_{p_1},x_{p_2}) = p(a_{p_1}=0,a_{p_2}=0|x_{p_1},x_{p_2})$ is the probability of parties p_1 and p_2 obtaining outcomes 0 when performing measurements x_{p_1} and x_{p_2}, and the single-party probabilities are defined analogously.

Note that Eq. (S1), despite being obtained from a two-output distribution, involves probabilities of only a single measurement, namely that denoted with the label 0. This indicates that the characterization provided by $\mathcal{O}_1$ is reasonably weak, and it does not exploit all the structure in the distribution. A consequence is that one can observe violations produced by a single source distributing classical bits to the parties, since any no-input distribution can be generated in this way. In fact, the GHZ distribution $p_{\text{GHZ}}(a_1,\ldots,a_6) = \frac{1}{2}$ if $a_1 = \cdots = a_6$ achieves a value of $\mathcal{W}_1 = -0.1393$.

One can obtain a stronger witness, that exploits the full information available, by increasing the level of the hierarchy of semidefinite programs. The next level that can be run with standard computing resources is the commonly known as level 1 + AB, defined by the subset of $\mathcal{O}_2$ given by $\mathcal{O}_{1+\text{AB}} := \{\mathbb{1}\}\cup\{A_p^{i,j}\}\cup\{A_p^{i,j}A_{p'}^{i',j'}\}_{p'\neq p}$. Requiring that the corresponding matrix, $\Gamma^{1+\text{AB}}$ (which has size 697×697) is positive semidefinite (taking ~ 470 seconds) reveals that this is impossible for $v \gtrsim 0.3887$. The associated witness is excessively large and complex, and thus we store it in machine-readable form in the computational appendix [62].

Witnesses of network structure for no-input distributions

In this section we show the analysis of the compatibility of no-input distributions generated in the setup of Fig. 1(b-i) in the main text with the network of Fig. 1(b-iii). The results for ideal, noiseless distributions are shown in Fig. S1, and those for the experimental data of Ref. [41] are shown in Fig. S2. In the vertical axes we write the (noiseless) distributions used to construct the corresponding witnesses, and in the horizontal axes we write the distribution that is evaluated on each of them. The distributions are obtained by performing each of the possible sets of measurements in $\{X,Z\}^{\times 6}$ at the rightmost ports in Fig. 1(b-i) when to the quantum state resulting from using maximally entangled states at the sources (leftmost ports) of the setup. In the figures, a blank row indicates that the corresponding distribution was not detected to be incompatible with the inflation considered, so no witness could be extracted. Figures 3a, 3b in the main text correspond to the bottom-right corners of Figs. S1, S2. Then, Fig. S3 contains the evaluations of the witnesses that detect as incompatible the largest amount of empirical distributions, in the empirical

distributions that are detected, as a function of the number of datapoints used for estimating $p(a_1, \ldots, a_6)$. All the witnesses are obtained by running the semidefinite programs corresponding to the second-order quantum inflation depicted in Fig. 2 in the main text and the NPA level $1 + \mathrm{AB}$. For the case of distributions without inputs, these lead to matrices $\Gamma^{1+\mathrm{AB}}$ of size 185×185, whose positivity can be determined in ~ 5.5 seconds.

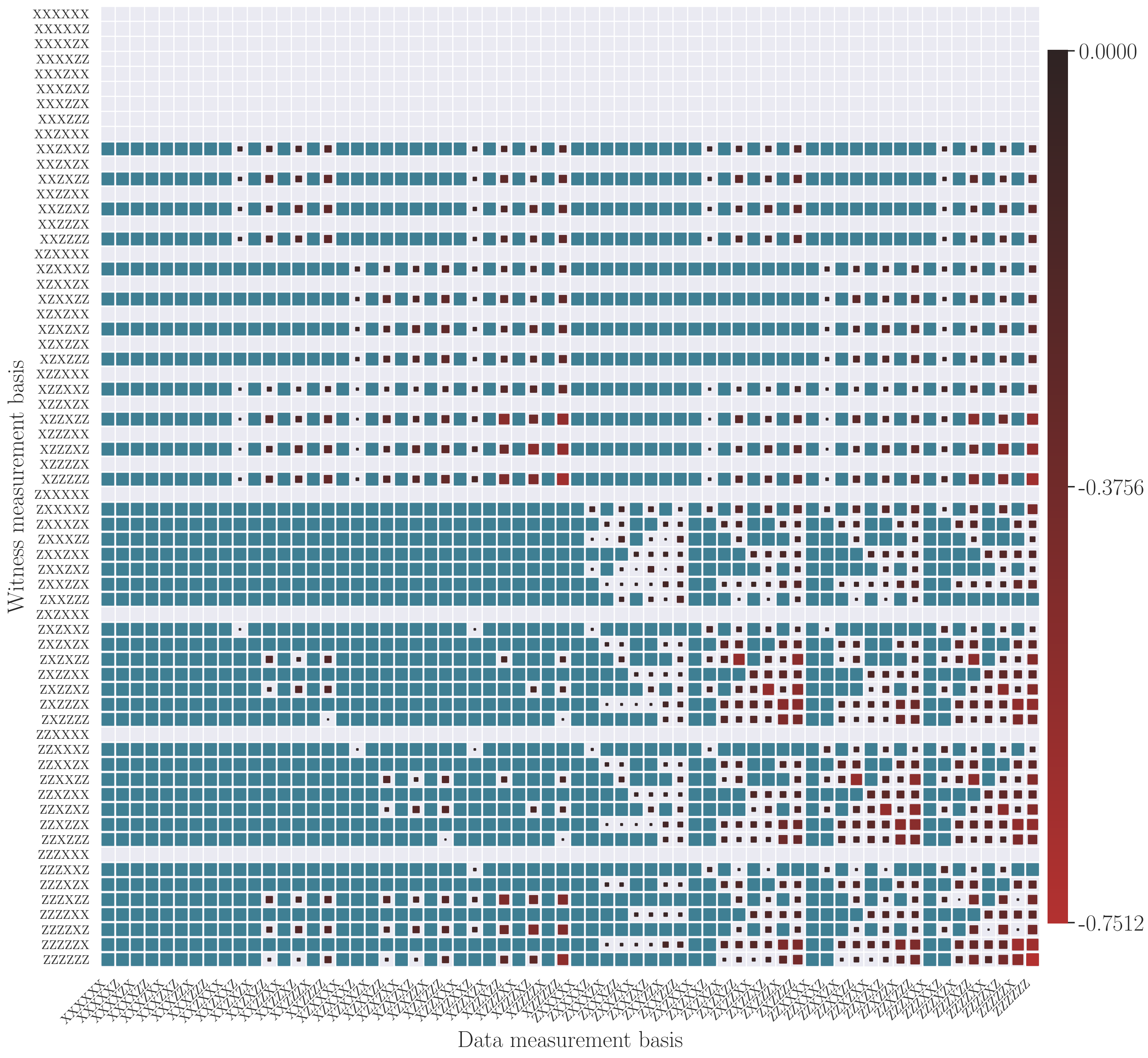

FIG. S1. Analysis of the compatibility of ideal no-input distributions. The indexing of the rows and columns denotes the measurement operators that are used to generate a no-input probability distribution according to Eq. (3) in the main text. Using the procedure described in the text, the distributions denoted by the rows are found to be incompatible with realizations in the network of Fig. 1(b-iii) in the main text, each one producing a witness of incompatibility. Then, each witness is evaluated on all distributions denoted by the columns, producing the figures where each cell represents the evaluation of the witness obtained from the distribution in the row in the distribution in the column. The blue cells denote distributions that are not detected by a particular witness, i.e. those that evaluate to a positive value. The empty rows denote ideal distributions that are not detected to be incompatible with the inflation used. The size and the color of the red squares denote the strength of the detection for distributions witnessed to be incompatible.

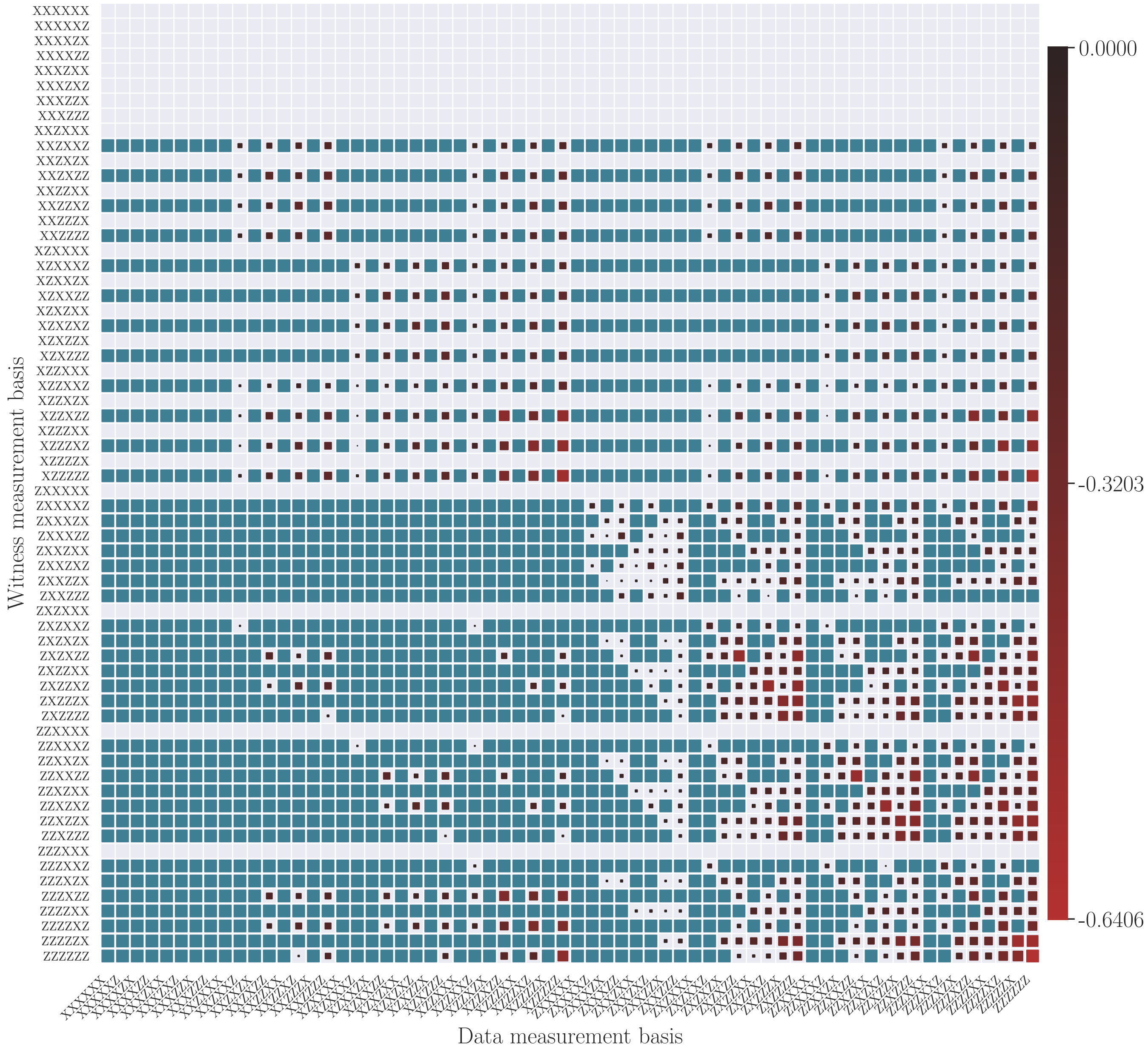

FIG. S2. Analysis of the compatibility of empirical no-input distributions created from the data of Ref. [41]. The indexing of the rows and columns denotes the measurement operators that are used to generate a no-input probability distribution according to Eq. (3) in the main text. Using the procedure described in the text, the distributions denoted by the rows are found to be incompatible with realizations in the network of Fig. 1(b-iii) in the main text, each one producing a witness of incompatibility. Then, each witness is evaluated on all distributions denoted by the columns, producing the figures where each cell represents the evaluation of the witness obtained from the distribution in the row in the distribution in the column. The blue cells denote distributions that are not detected by a particular witness, i.e. those that evaluate to a positive value. The empty rows denote ideal distributions that are not detected to be incompatible with the inflation used. The size and the color of the red squares denote the strength of the detection for distributions witnessed to be incompatible.

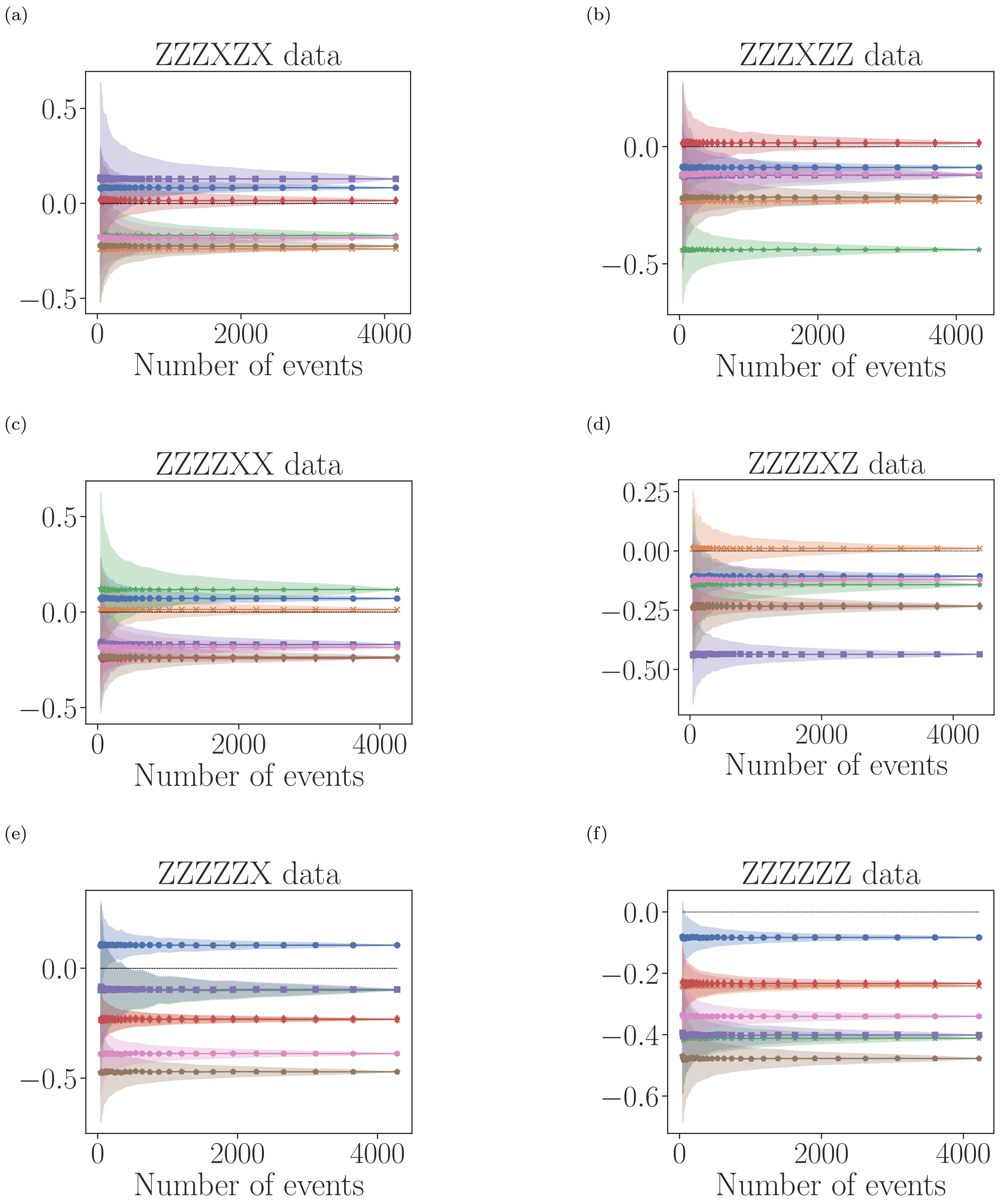

FIG. S3. Illustration of evaluation of the structure witnesses in the experimental data of Ref. [41] for the no-input distributions as a function of the amount of experimental data used in the evaluation. The witnesses evaluated correspond to the following measurement bases: (blue circles) $ZZXXXZ$, (orange crosses) $ZZXXZX$, (green stars) $ZZXXZZ$, (red diamonds) $ZZXZXX$, (purple squares) $ZZXZXZ$, (brown pentagons) $ZZXZZX$, (pink hexagons) $ZZXZZZ$. The minimum value achievable by quantum distributions generated in the network of Fig. 1(b-iii) in the main text is lower bounded by 0 in all cases (the dashed black line), so evaluating to a negative number by a given distribution is a witness that such distribution cannot be generated in Fig. 1(b-iii). In the horizontal axis we denote the amount of all datapoints, chosen at random, used for computing the witness. Error bars correspond to five standard deviations over 100 repetitions.

CERTIFICATION IN THE TRIDENT GRAPH

In addition to the experimental setup of Ref. [41], we have analyzed an additional realization. This is the one present in [40], known as the Trident graph, and depicted in Fig. 1(c) in hte main text. In contrast with the previous one, the central photon source does not distribute Bell states, but separable states, and in exchange there is a third fusion gate that acts upon photons 3 and 4. Therefore, the quantum distributions generated in it take the form

$$p(a_1,\ldots,a_6) = \mathrm{Tr}\left[U_{34}\otimes U_{23}\otimes U_{45}\left(\phi^+_{12}\otimes\psi_3\otimes\psi_4\otimes\phi^+_{56}\right)U^\dagger_{45}\otimes U^\dagger_{23}\otimes U^\dagger_{34}\cdot\left(\Pi_{a_1}\otimes\cdots\otimes\Pi_{a_6}\right)\right], \tag{S2}$$

where $\psi = (|0\rangle + |1\rangle)/\sqrt{2}$.

The associated network, obtained by connecting the sources with all the parties causally connected to them, is depicted in Fig. 1(c-iii) in the main text, reproduced in Fig. S4(a). In this case, note that the source S_1 distributes systems to parties A_1, A_2, A_3 and A_4, and the source S_2 distributes systems to parties A_2, A_3 and A_4. Since in quantum inflation the dimension of the systems is not constrained, one can without loss of generality absorb the source S_2 into S_1, and the source S_3 into S_4. Therefore, one can instead contrast against the network in Fig. S4(b) without loss of generality. In the following, we show results for the analysis of no-input and two-input distributions, in an analogous manner to the exposition in the main text.

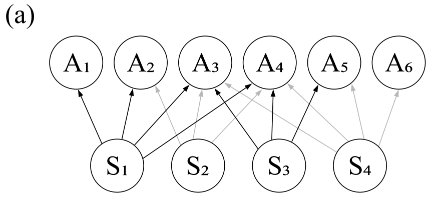

FIG. S4. Closest network to the trident experiment of [40]. (a) is a reproduction of Fig. 1(b-iii) in the main text. Since the dimension of the systems is unrestricted in inflation, the sources S_2 and S_3 in the naive network of (a) can be absorbed in sources S_1 and S_4, respectively, leading to the network in (b).

No-input distributions

We begin by obtaining and analyzing the witnesses of no-input distributions. Reference [40] reports data for a total of 417 choices of measurements in $\{X, Y, Z\}^{\times 6}$. We thus assess the compatibility of all the corresponding distributions with a realization in the network of Fig. S4(b), i.e., of the form

$$p(a_1,\ldots,a_6) = \mathrm{Tr}\left[(\rho_{S_1}\otimes\rho_{S_4})\cdot(\Pi_{a_1}\otimes\cdots\otimes\Pi_{a_6})\right]. \tag{S3}$$

For doing so, we consider the second-order quantum inflation of Fig. S4(b). The fact that this network contains one fewer source than the inflation of the network in Fig. 1(b-iii) allows us to consider higher levels of the associated NPA hierarchy. More concretely, we use the generating set $\mathcal{O}_{1+\mathrm{AB}+\mathrm{ABC}} := \{\mathbb{1}\}\cup\{A^{i,j}_p\}\cup\{A^{i,j}_p A^{i',j'}_{p'}\}_{p'\neq p}\cup\{A^{i,j}_p A^{i',j'}_{p'} A^{i'',j''}_{p''}\}_{p\neq p'\neq p''\neq p}$, which produces a matrix $\Gamma^{1+\mathrm{AB}+\mathrm{ABC}}$ of size 473×473, taking ~ 80 seconds to determine its positivity. The corresponding semidefinite programs only identify 22 distributions as not admitting a realization in terms of Eq. (S3). These are the ones depicted in the vertical axes in the plots of Fig. S5. When using noisy states of visibility v in the sources, the incompatibility can be detected, depending on the particular witness, until visibilities ranging from $v = 0.6298$ (for measurement bases $YYXXYY$, $YYXYYY$, $YYYZYY$, $YYYZZX$, $YYZZYY$, $YYZZZX$, $ZXXXZX$, $ZXYZYY$, $ZXZZYY$, and $ZXZZZX$) to $v = 0.8409 \sim 2^{-1/4}$ (for measurement bases $YYYXYY$, $YYYYYY$, $YYZXYY$, $YYZXZX$, $YYZYYY$, $YYZYZX$, $ZXYYZX$, $ZXZXYY$, and $ZXZYYY$).

As is shown in Fig. S5b, the empirical data is still in good agreement with the theory and several distributions can be witnessed as incompatible by many of the witnesses. However, out of all the 417 empirical distributions, the only ones that are detected by the witnesses are those in the horizontal axes in Fig. S5. Moreover, the relative magnitudes of the experimental to theoretical evaluations is smaller in this case than in that of the data from Ref. [40] showcased in the main text. These two phenomena may be explained by the difference in acquisition times and number of counts obtained in each experiment. Yet, the fact that it is possible to demonstrate that the empirical distributions cannot

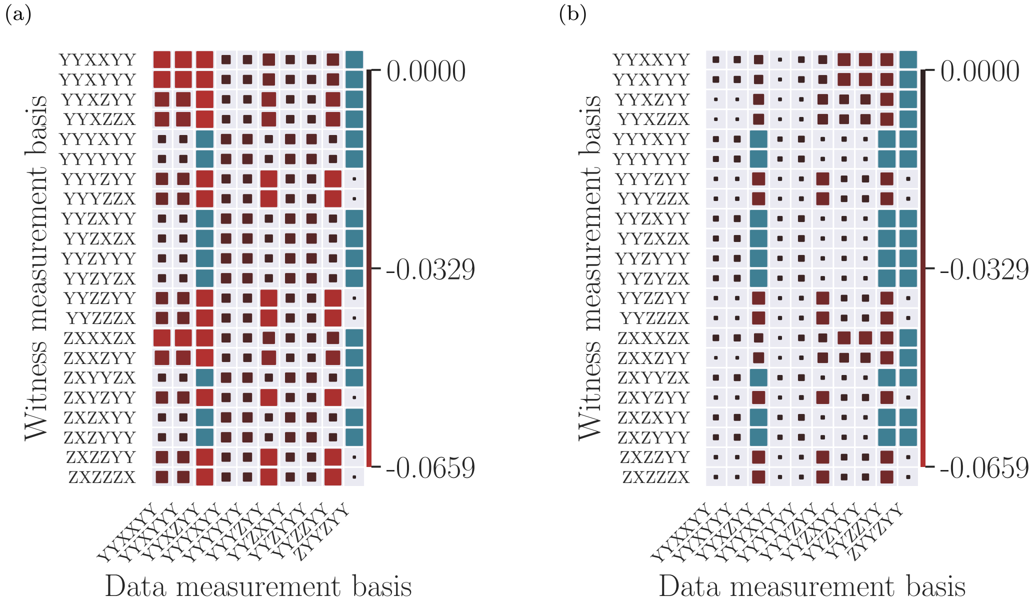

FIG. S5. (a) Theoretical predictions and (b) experimental results for the witnesses of incompatibility with a realization of the form of Eq. (S3). The indexing of the rows and columns denotes the measurement operators that are used to generate a no-input probability distribution according to Eq. (S2). Using the procedure described in the text, the distributions denoted by the rows are found to be incompatible with realizations in the network of Fig. S4(b) (i.e., incompatible with realizations of the form of Eq. (S3)), each one producing a witness of incompatibility. Then, each witness is evaluated on all distributions denoted by the columns, producing the figures where each cell represents the evaluation of the witness obtained from the distribution in the row in the distribution in the column. The blue cells denote distributions that are not detected by a particular witness, i.e. those that evaluate to a positive value. The empty rows denote ideal distributions that are not detected to be incompatible with the inflation used. The size and the color of the red squares denote the strength of the detection for distributions witnessed to be incompatible. The plotted bases in the horizontal axes are the only ones for which the corresponding empirical distributions are witnessed as incompatible.

be generated in the network of Fig. S4(b) motivates the research on conference key agreement protocols, such as those tested in Ref. [40], using the measurement bases in Fig. S5.

Binary-input distributions

For the case when the parties have a choice between two binary-outcome measurements to perform, the data available in Ref. [40] allows to consider three simple cases, namely when all parties choose their measurements from the same pair, which can be $X-Y$, $X-Z$ or $Y-Z$. We assess whether the corresponding theoretical distributions (i.e., in the form of Eq. (S2), when generalized to the parties admitting inputs) admits a realization in the form Eq. (S3) by considering the corresponding second-order inflation, and the corresponding generating set $\mathcal{O}_{1+\mathrm{AB}}$, that leads to assessing the positivity of a matrix $\Gamma^{1+\mathrm{AB}}$ of size 449×449 (taking ~ 155 seconds to do so). All distributions are witnessed to be incompatible (down to visibilities of $v=0.8421$ when considering noisy states), and each of them

produces one witness, namely

$$
\begin{aligned}
\mathcal{W}_{XY}^{1+\mathrm{AB}} = & p_{AB}(00)p_C(1) - p_{ABC}(001) + p_C(1)p_{EF}(00) - p_{CEF}(100) \\
& + \frac{1}{\sqrt{2}}[p_{AB}(00)(p_A(0)+p_B(0)-p_{AB}(00)) + p_{EF}(00)(p_E(0)+p_F(0)-p_{EF}(00))] \\
& + \frac{1}{2}[p_{AC}(01) - p_A(0)p_C(1) + p_{BC}(01) - p_B(0)p_C(1) + p_{CE}(10) - p_C(1)p_E(0) + p_{CF}(10) - p_C(1)p_F(0)] \\
& + \frac{1}{2\sqrt{2}}[p_C(1) - p_C(1)^2 - p_{AB}(00) - p_A(0)p_B(0) - p_E(0)p_F(0) - p_{EF}(00)] \\
& + \frac{1}{4\sqrt{2}}[p_A(0) + p_B(0) + p_E(0) + p_F(0) - p_A(0)^2 - p_B(0)^2 - p_E(0)^2 - p_F(0)^2],
\end{aligned}
\tag{S4}
$$

$$
\begin{aligned}
\mathcal{W}_{XZ}^{1+\mathrm{AB}} = & p_{AB}(01)(p_A(0)+p_B(1)) + p_{EF}(01)(p_E(0)+p_F(1)) - p_{AB}(01)^2 - p_{EF}(01)^2 \\
& + 0.471[(p_{AB}(01)+p_{EF}(01))(p_{CD}(10)+p_C(1)+p_D(0)) \\
& \qquad - p_{ABCD}(0110) - p_{ABC}(011) - p_{ABD}(010) - p_{CDEF}(1001) - p_{CEF}(101) - p_{DEF}(001)] \\
& - \frac{1}{2}[p_{AB}(01) + p_A(0)p_B(1) + p_{EF}(01) + p_E(0)p_F(1)] \\
& + 0.236[p_{ACD}(010) + p_{AC}(01) + p_{AD}(00) - p_A(0)(p_C(1)+p_D(0)+p_{CD}(10)) \\
& \qquad + p_{BCD}(110) + p_{BC}(11) + p_{BD}(10) - p_B(1)(p_C(1)+p_D(0)+p_{CD}(10)) \\
& \qquad + p_{CDE}(100) + p_{CE}(10) + p_{DE}(00) - (p_{CD}(10)+p_C(1)+p_D(0))p_E(0) \\
& \qquad + p_{CDF}(101) + p_{CF}(11) + p_{DF}(01) - (p_{CD}(10)+p_C(1)+p_D(0))p_F(1)] \\
& + \frac{1}{4}[p_A(0) + p_B(1) + p_E(0) + p_F(1) - p_A(0)^2 - p_B(1)^2 - p_E(0)^2 - p_F(1)^2] \\
& + 0.388 p_{CD}(10) \\
& + 0.055[p_C(1) - p_C(1)^2 + p_D(0) - p_D(0)^2 - p_{CD}(10)^2] \\
& - 0.111[p_{CD}(10)(p_C(1)+p_D(0)) + p_C(1)p_D(0)],
\end{aligned}
\tag{S5}
$$

$$
\begin{aligned}
\mathcal{W}_{YZ}^{1+\mathrm{AB}} = & p_{AB}(00)p_D(1) - p_{ABD}(001) + p_D(1)p_{EF}(00) - p_{DEF}(100) \\
& + \frac{1}{\sqrt{2}}[p_{AB}(00)(p_A(0)+p_B(0)-p_{AB}(00)) + p_{EF}(00)(p_E(0)+p_F(0)-p_{EF}(00))] \\
& + \frac{1}{2}[p_{AD}(01) - p_A(0)p_D(1) + p_{BD}(01) - p_B(0)p_D(1) + p_{DE}(10) - p_D(1)p_E(0) + p_{DF}(10) - p_D(1)p_F(0)] \\
& + \frac{1}{2\sqrt{2}}[p_D(1) - p_D(1)^2 - p_{AB}(00) - p_A(0)p_B(0) - p_{EF}(00) - p_E(0)p_F(0)] \\
& + \frac{1}{4\sqrt{2}}[p_A(0) + p_B(0) + p_E(0) + p_F(0) - p_A(0)^2 - p_B(0)^2 - p_E(0)^2 - p_F(0)^2].
\end{aligned}
\tag{S6}
$$

Note that Eqs. (S4) and (S6) are essentially the same inequality with the role of parties C and D exchanged. Moreover, as in the case with the setup of Ref. [41], the inequalities are effectively single-input inequalities.

A straightforward calculation shows that each witness is violated by the corresponding distribution, but not by those corresponding to other sets of inputs. The evaluations on the experimental data are shown in Table I. None of the evaluations give a clear violation, and thus one needs to consider higher levels in the semidefinite programming hierarchy in order to obtain witnesses that are violated by the empirical data.

[68] A. Pickston, F. Graffitti, P. Barrow, C. L. Morrison, J. Ho, A. M. Brańczyk, and A. Fedrizzi, Opt. Express **29**, 6991 (2021).
[69] D. E. Browne and T. Rudolph, Phys. Rev. Lett. **95**, 010501 (2005).

	XY	XZ	YX	YZ	ZX	ZY
XY	0.1687 ± 0.0240	0.3720 ± 0.0278	0.1759 ± 0.0251	-0.0137 ± 0.0294	0.1164 ± 0.0320	0.1125 ± 0.0279
XZ	0.2045 ± 0.0231	0.1929 ± 0.0266	0.2000 ± 0.0227	0.1978 ± 0.0281	0.1910 ± 0.0284	0.2065 ± 0.0269
YZ	0.1809 ± 0.0271	0.3695 ± 0.0275	0.1779 ± 0.0268	-0.0050 ± 0.0317	0.1105 ± 0.0293	0.1103 ± 0.0283

TABLE I. Evaluations of the witnesses in Eqs. (S4)-(S6) (in the rows) in the experimental data of [40] (in the columns). The order of the bases in the first row determines which basis corresponds to measurements 0 and 1 in Eqs. (S4)-(S6).
